# Characterization of Silver Conductive Ink Screen-Printed Textile Circuits: Effects of Substrate, Mesh Density, and Overprinting

**DOI:** 10.3390/ma17194898

**Published:** 2024-10-06

**Authors:** Hyobin Im, Jung-Sim Roh

**Affiliations:** 1 Culture Technology Research Center, Sangmyung University, Seoul 03016, Republic of Korea; hyobin25@nate.com; 2 Department of Fashion and Textiles, Sangmyung University, Seoul 03016, Republic of Korea

**Keywords:** screen printing, electronic textiles, textile circuits, conductive ink, smart wearables, screen-printing processes, cotton fabric substrates, polyester fabric substrates, overprinting

## Abstract

This study explores the intricate interaction between the properties of textile substrates and screen-printing parameters in shaping fabric circuits using silver conductive ink. Via analyzing key variables such as fabric type, mesh density, and the number of overprinted layers, the research revealed how the porous structure, large surface area, and fiber morphology of textile substrates influence ink absorption, ultimately enhancing the electrical connectivity of the printed circuits. Notably, the hydrophilic cotton staple fibers fabric effectively absorbed the conductive ink into the fabric substrate, demonstrating superior electrical performance compared with the hydrophobic polyester filament fabric after three overprinting, unlike the results observed after a single print. As mesh density decreased and the number of prints increased, the electrical resistance of the circuit gradually reduced, but ink bleeding on the fabric surface became more pronounced. Cotton fabric, via absorbing the ink deeply, exhibited less surface bleeding, while polyester fabric showed more noticeable ink spreading. These findings provide valuable insights for improving screen printing technology for textile circuits and contribute to the development of advanced fabric circuits that enhance the functionality of smart wearable technology.

## 1. Introduction

Electronic textiles, which integrate electrical and electronic functions into fibers, are used to develop wearable products for various applications such as sports, healthcare, and safety. To develop smart wearable clothing, flexible fiber circuit manufacturing technology is essential. Flexible fiber circuits are technologies that provide electrical conductivity while maintaining the flexibility of the fibers. These circuits can be constructed using conductive fibers integrated into various fabric structures during manufacturing processes, such as weaving [1,2], knitting [3,4], and transmission cables [5,6]. Additionally, they can be created by embroidering conductive threads [7,8] or patterning electronic circuits onto fabrics using conductive ink printing [9,10,11,12]. Weaving and knitting methods have high durability because circuits are formed during fabric production using separate conductive threads, but they make free circuit design difficult. On the other hand, embroidery and printing methods offer the advantage of being able to freely create circuits with conductive threads or conductive ink on various base fabrics. Methods using conductive fibers require the prior development of conductive threads, necessitating expertise and practical know-how in textile technology. Additionally, because they involve the use of textile production facilities, they are difficult for non-experts to access. On the other hand, the method of printing conductive materials onto the fiber surface is accessible to anyone without specialized skills and allows for quick implementation of various circuit patterns. This approach is being utilized for the development of various smart wearables, including heating elements for heaters [13,14], wearable antennas [15,16,17,18], motion sensors [19], sensors for monitoring bio-signals like ECG or EMG [20,21,22], electrode fabrication [23], and location tracking devices [24].

The primary printing methods are inkjet printing and screen printing. Inkjet printing is a non-contact method where ink is ejected from a nozzle, which moves back and forth along the x and y axes. This method uses less ink than screen printing, allows for automated processes, and enables the printing of digital models. However, it has a slower printing speed and potential issues such as nozzle clogging. On the other hand, screen printing, which creates circuits via applying conductive ink through the mesh openings of a screen onto the substrate, has the advantage of being able to quickly produce a large volume of diverse and complex circuit patterns at a low cost, without being significantly constrained by the type of substrate. In screen printing, the amount of ink transferred to the substrate depends on many variables such as ink viscosity, mesh density, and squeegee speed, and the substrate’s characteristics affect how the ink is absorbed. Therefore, it is crucial to establish a process optimized for the desired circuit precision and electrical performance.

Unlike general non-porous substrates, fabrics formed using weaving or knitting fibers have numerous micropores and uneven surfaces, which can be disadvantageous for the uniform fabrication of circuit patterns. To reduce the impact of the substrate fabric, a smooth interface layer can be applied to the fabric surface to implement conductive tracks [25]. However, this method can negatively affect the fabric’s breathability and feel and is not suitable for large-area printed circuits, making it difficult to generalize. To implement printed circuits directly on fabric, it is essential to first understand how the characteristics of the substrate fabric and the conditions of the conductive screen-printing process affect the formation of textile circuits [15,26,27]. In this study, we set hydrophilic cotton fabric and hydrophobic polyester fabric, which are representative clothing materials, as representative substrate fabrics, and quantitatively evaluated the appearance characteristics and electrical performances depending on various process conditions of screen-printed circuit patterning using conductive ink, thereby characterizing the production aspect of fiber-based printed circuits. The results of this study can serve as a guideline for various smart-wearable developers to estimate the screen-printing process conditions optimized for the fabric properties they will use.

## 2. Materials and Methods

### 2.1. Materials

For the development of textile printed circuits, we used screen-printing conductive ink with excellent conductivity and adhesion, which is employed in various applications (Henkel Co. LOCTITE EDAG 479SS E&C, Korea Chemical Co., Ltd., Hwaseong, Republic of Korea) (Table 1).

As substrate materials, we selected plain structure fabrics of cotton and polyester, which are commonly used in clothing. For performance comparison, we also prepared polyester film (Table 2).

A screen-printing mesh is used to properly control the amount of conductive ink applied to the substrate and to pattern the shape of the circuit. The cover factor of the mesh is one of the key factors that affects the ink transfer and the precision of the print. In this study, experiments were conducted with three types of screen meshes (70G, 120G, 200G) with different cover factors to examine the effect of the screen mesh on the substrate fabric. The mesh number represents the number of meshes per inch in either the horizontal or vertical direction, indicating mesh density. A lower mesh density corresponds to a smaller cover factor, which allows more ink to pass through the screen mesh and be transferred onto the substrate fabric. Conversely, a higher mesh density with a larger cover factor prevents excessive ink from being applied to the substrate. This study will examine the impact of mesh density (low, medium, high) on the characteristics of the substrate fabric and the overprinting printing process.

### 2.2. Circuit Design

Table 3 shows five types of circuits designed for the experiment. These include a basic design (#1) with a length of 60 mm and a width of 12 mm, a circuit with twelve parallel 1 mm wide straight lines spaced 1 mm apart (#2), a circuit with six parallel 2 mm wide straight lines spaced 2 mm apart (#3), a circuit with six parallel 1 mm wide embattled lines spaced 1 mm apart (#4), and a circuit with three parallel 2 mm wide embattled lines spaced 2 mm apart (#5). The 5 mm sections at both ends of each circuit were used as electrodes for measuring electrical resistance.

### 2.3. Screen-Printing Process

**Figure 1:**
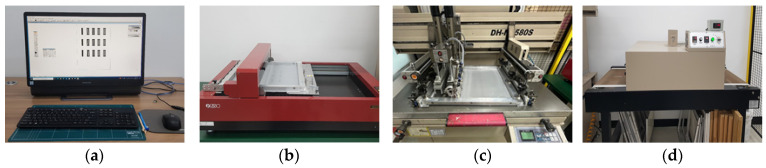
Screen printing process: (**a**) control system, (**b**) digital screen making, and (**c**) screen printing, (**d**) IR drying.

The designed circuit patterns were transferred to an illustration program, arranged according to the printing direction (Figure 1a), and then output onto a mesh attached to a mesh frame using a computer screen maker (GOCCOPRO QS2536, Riso Korea Co., Ltd., Seongnam, Republic of Korea) (Figure 1b). The mesh frame with the printed circuit was mounted onto a screen printer (SP-5080EP, East Core Co., Ltd., Seoul, Republic of Korea). The substrate fabric was placed on the worktable and fixed in place using vacuum suction. The silver conductive ink was preconditioned at room temperature for 1 h and then stirred with a metal spatula for 1 min. The silver conductive ink was applied at the starting edge of the mesh, and printing was performed with a scan speed of 0.06 m/s and a squeegee angle of 25° (Figure 1c). For overlapping prints, the process was repeated after allowing the previous layer to air-dry for 10 min. The printed samples were dried in a convection oven at 93 °C for 15 min (Figure 1d). In this study, printed circuit samples were produced in triplicate for each substrate fabric, varying the mesh density (70G, 120G, 200G) and the number of overprinting (1, 2, 3) for the five circuit patterns.

### 2.4. Measurement

To investigate the printing characteristics and electrical properties of silver conductive ink screen-printed circuits according to substrate type and processing conditions, the appearance of the fabricated samples was observed and electrical resistance was measured. The surface and cross-section of the printed circuits were photographed, and the thickness of the conductive layer was measured to assess the printing quality of the screen-printed circuits. The surface of the printed circuits was imaged using an SV-55 video microscope system (SOMETECH Inc., Seoul, Republic of Korea), and the entire printed circuit was scanned with a DCP-T300 (Brother Industries. Ltd., Guri, Republic of Korea) to observe the surface where the silver conductive ink layer was formed. The cross-sectional Scanning Electron Microscope (SEM, Crossbeam 550L, Carl Zeiss Co., Ltd., Seoul, Republic of Korea) images of the printed circuits and measurements of the circuit thickness were used to examine the formation of the conductive layer.

Additionally, when printing with conductive ink on fabric, bleeding occurs at the pattern edges, causing the circuit pattern to increase in both width and length. The changes in width and length were measured at 10 specific locations on the printed circuit, and the average values were compared.

To verify the connectivity performance of the printed circuits, the electrical resistance of the screen-printed circuits was measured using a low-resistance meter (Agilent 4338B, ATM Inc., Seoul, Republic of Korea). To minimize contact resistance at the electrode area during the measurement, a metal wire was placed across the center of the electrode section of the printed circuit, attached with conductive tape, and a consistent high pressure was applied using a pressure clamp. The wire was then clamped with a 4-probe clip for measurement.

## 3. Results and Discussion

### 3.1. Printing Appearance Characteristics

#### 3.1.1. Surface Appearance

Table 4 and Table 5 show the surface microscope images and scan images, respectively, of the circuit (#1) screen-printed with silver conductive ink on three different types of substrate materials. The formation of the conductive layer varied depending on the characteristics of the fabric used as the substrate and the printing process conditions. Since the amount of ink transferred to the substrate through the mesh varies with the mesh cover factor, the mesh density must be chosen to ensure that details are properly expressed and the conductive ink layer is formed accurately. In this study, it was observed that, regardless of the substrate type, the higher the mesh density and the number of overprint count, the clearer the boundaries of the screen-printed circuits became.

**Figure 2:**
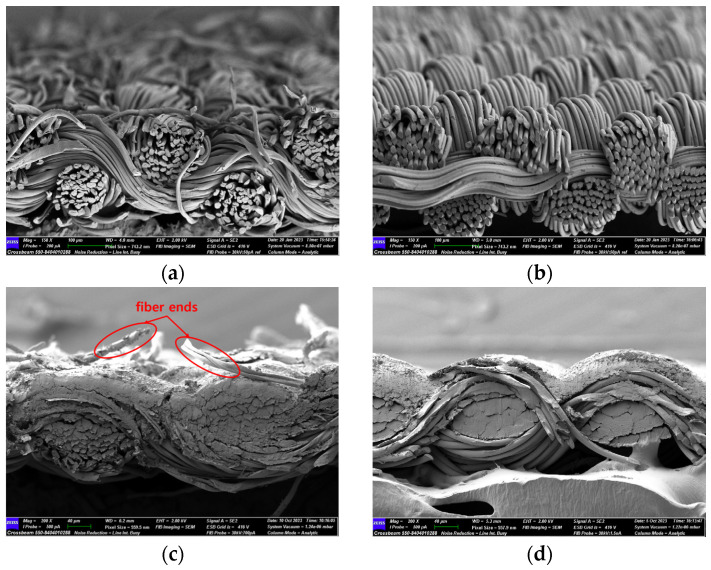
SEM images of the cross-section of substrate fabrics: (**a**) cotton fabric (150×), (**b**) polyester fabric (150×), (**c**) printed circuit on cotton fabric (#1, 120G, single screen-printed, 200×), and (**d**) printed circuit (#1) on polyester fabric (#1, 120G, single screen-printed, 200×).

There were differences in the surface appearance based on the substrate fabric type and printing process conditions. With a single print, using a 200G high-density mesh, the formation of the conductive layer was inadequate for both cotton and polyester fabric substrates. Cotton fabric, being a typical hydrophilic fiber material with a large surface area covered by staple fibers arranged obliquely from the surface to the back of the fabric (Figure 2a), absorbed a significant amount of the conductive ink through the mesh into its hydrophilic fibers. As a result, the black color of the cotton fabric clearly showed through the silver conductive layer with a single print at all mesh densities. In contrast, the polyester fabric formed a relatively well-defined conductive layer on the surface compared with the cotton fabric substrate because its filament yarns are arranged parallel to the fabric surface (Figure 2b).

Table 6 clearly shows that the silver conductive layer on the fabric substrate became thicker with decreasing mesh density and an increasing number of prints. The photographs in Table 5 and Table 6 show that a solid silver conductive layer was formed for all types of substrate fabric and mesh conditions with two or more overprints.

#### 3.1.2. Thickness of Conductive Layer

**Figure 3:**
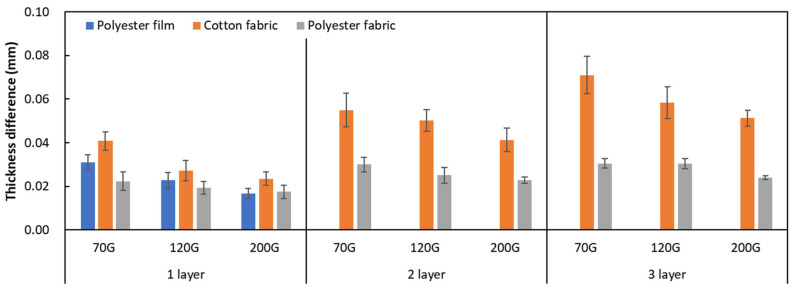
Thickness of conductive layer (#1).

To determine the degree of conductive layer formation on the substrate fabric according to mesh density and number of overprints, the thickness of the printed circuit (#1) was measured. The conductive layer thickness in Figure 3 is defined as the increase in thickness compared to the substrate fabric thickness before printing and is the average of three measurements.

Figure 3 shows that the thickness of the printed circuit increased with decreasing mesh density and an increasing number of prints. Unlike the polyester film substrate, the cotton and polyester fabric substrates showed different patterns in the thickness of the conductive layer due to their chemical compatibility with the conductive ink, the fiber pore structure, and the large surface area. For the polyester film substrate, the thickness of the conductive circuit was measured to be 0.0309 mm, 0.0228 mm, and 0.0167 mm for the 70G, 120G, and 200G mesh processes, respectively. For the cotton fabric substrate, the thickness was 0.0409 mm, 0.0272 mm, and 0.0236 mm, respectively, and for the polyester fabric substrate, the thickness was 0.0223 mm, 0.0194 mm, and 0.0176 mm, respectively. Since polyester fabric is hydrophobic, the conductive ink gradually penetrated into the pores of the fibers after squeegeeing and drying, resulting in a numerically lower thickness of the conductive layer compared with polyester film, where the ink remained only on the surface. In contrast, the cotton fabric substrate exhibited a significant increase in the thickness of the printed circuit. This is because the conductive ink was rapidly and significantly absorbed into the fabric as soon as it touched it due to the hydrophilicity of cotton fibers and the structural properties of the spun yarn. The spun yarn, which was made via twisting spun fibers (Figure 2a), transported moisture more effectively through the thickness direction of the fabric than the filament fibers fabric (Figure 2b). As a result, the cotton fabric substrate absorbed more conductive ink in each printing pass, resulting in a significant increase in the thickness of the printed circuit compared with the polyester fabric substrate.

#### 3.1.3. Detail Resolution

**Figure 4:**
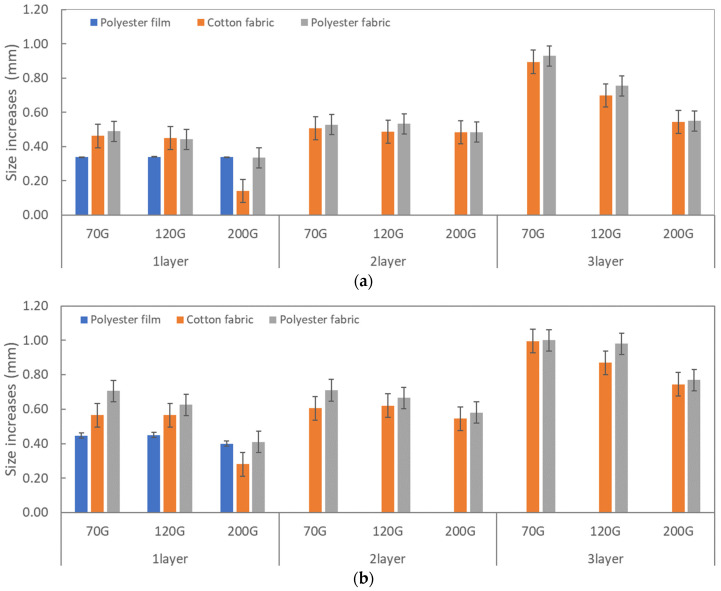
Size increases in width (**a**) and length (**b**) directions between the screen-printed circuits and original circuit designs.

The size variation caused by bleeding was observed depending on the characteristics and processing conditions of the fabric used as a substrate material (Table 7). To evaluate the degree of this variation, the increase in size was measured for circuit pattern #4, which consisted of a 1 mm embattled line, relative to the original design dimensions. Figure 4 presents the average values of the size increase in both the width and length directions of the printed circuit, as measured at 10 different locations, as shown in Table 7.

In the case of fabric substrates, the size increase in the printed circuit due to bleeding was greater compared with the polyester film substrates, owing to the effects of absorption and wicking inherent to the fabric’s characteristics. The size increase was significantly influenced by the mesh density. Lower mesh density resulted in greater exposure to ink, leading to a more pronounced increase in the size of the printed circuit. The bleeding was more pronounced in the length direction, which corresponds to the squeegee’s movement direction, than in the width direction.

The bleeding of the printed circuit was more pronounced on polyester fabric compared with cotton fabric. The higher the surface roughness of the fabric and the lower the density of the fabric, the higher the roughness of the printed lines. The roughness and density of the fabric affect the roughness of the printed lines [26]. This can be attributed to the fact that, as shown in the fabric images in Figure 2, the polyester filament fibers are horizontally aligned on the fabric surface, making it easier for the conductive ink to spread across the surface. The cotton fabric with a 200G mesh exhibited the least bleeding, which illustrates how a small amount of ink, delivered through the high-density mesh, is absorbed into the thickness of the cotton fabric.

As the overprinting process progressed, a sufficient amount of conductive ink was absorbed into the fabric, which significantly increased the size of the printed circuit. Therefore, when designing textile printed circuits using screen-printing techniques, the size increase in both the width and length directions due to the processing conditions must be taken into consideration.

### 3.2. Electrical Characteristics

**Figure 5:**
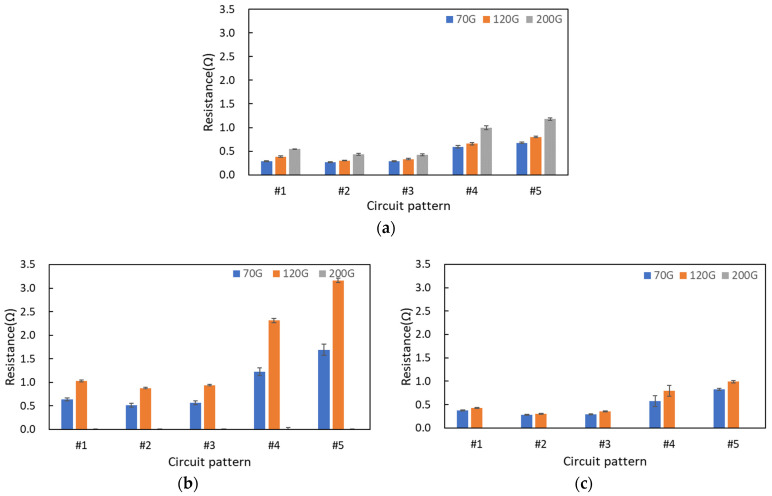
Electrical resistance of single screen-printed textile circuits depending on mesh density, substrate properties, and circuit pattern: (**a**) polyester film substrate, (**b**) cotton fabric substrate, and (**c**) polyester fabric substrate.

To evaluate the electrical performance of printed circuits based on the characteristics of the fabric substrates and processing conditions, the electrical resistance of screen-printed circuits was measured for five patterns (#1, #2, #3, #4, #5) across different substrate materials, mesh densities, and printing overprinting (Figure 5). It was observed that higher mesh density led to higher electrical resistance in the printed circuits, due to the reduced amount of ink transferred to the substrate. For the polyester film substrate, resistance values were measured in all printed circuits using 70G, 120G, and 200G mesh. However, for the 200G mesh density on cotton and polyester fabric substrates, the circuit formation was insufficient, and the electrical resistance was not measurable after a single print. For the basic circuit (#1) printed once on the polyester film substrate with 70G, 120G, and 200G mesh densities, resistance values of 291 mΩ, 386 mΩ, and 543 mΩ were measured, respectively. For the cotton fabric substrate, resistance values of 639 mΩ and 1027 mΩ were recorded with 70G and 120G meshes, respectively. For the polyester fabric substrate, resistance values of 374 mΩ and 431 mΩ were recorded with 70G and 120G meshes, respectively. The polyester fabric substrate exhibited electrical resistance values similar to those of the polyester film substrate. Although the cotton fabric substrate, which is hydrophilic, absorbed more ink immediately upon printing, resulting in a thicker printed circuit compared with the polyester fabric substrate, the electrical connectivity of the circuit was poor after a single print. As confirmed in the surface scan images of the printed circuit in Table 5, the resistance values were approximately twice as high when using 70G mesh and three times as high when using 120G mesh, compared with the hydrophobic polyester fabric substrate. Therefore, for single printing, it is recommended to use a medium or lower density mesh, and polyester fabric substrates were more advantageous than cotton fabric substrates in terms of circuit connectivity.

**Figure 6:**
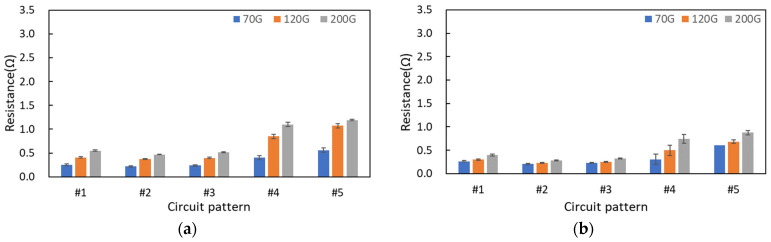
Electrical resistance of two overprinting screen-printed textile circuits dependent on mesh density, substrate properties, and circuit pattern: (**a**) cotton fabric substrate and (**b**) polyester fabric substrate.

**Figure 7:**
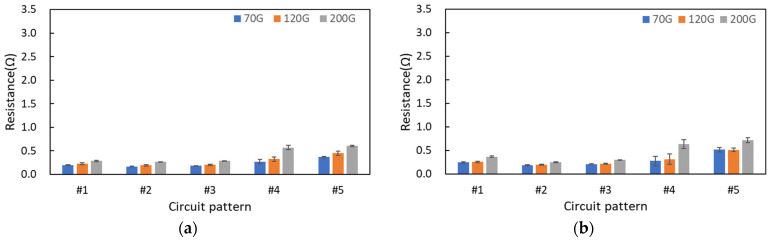
Electrical resistance of three overprinting screen-printed textile circuits dependent on mesh density, substrate properties, and circuit pattern: (**a**) cotton fabric substrate and (**b**) polyester fabric substrate.

To improve the electrical connectivity of textile printed circuits, screen printing was overprinted two (Figure 6) or three times (Figure 7). As the number of overprinting increased, lower mesh density resulted in lower electrical resistance. In the case of the circuit (#1) on the cotton fabric substrate with two overprints, the circuit resistance values were measured as 248 mΩ, 409 mΩ, and 550 mΩ for the 70G, 120G, and 200G processes, respectively. On the polyester fabric substrate, the resistance values were measured as 261 mΩ, 302 mΩ, and 397 mΩ, respectively. When printed twice, the cotton fabric substrate at a mesh density of 70G and the polyester fabric substrate at all mesh densities showed lower electrical resistance compared with the circuit with a single print. For the basic circuit (#1) with three prints, the resistance values on the cotton fabric substrate were measured as 191 mΩ, 222 mΩ, and 282 mΩ for the 70G, 120G, and 200G processes, respectively. On the polyester fabric substrate, the resistance values were measured as 245 mΩ, 256 mΩ, and 368 mΩ, respectively. The lower resistance of the printed circuit on the cotton fabric substrate compared with that on the polyester fabric substrate was due to the higher absorption of the conductive silver ink by the hydrophilic cotton yarn, which allowed the absorbed conductive material to be better electrically connected internally.

Previous studies [16,26] have suggested that synthetic filament fabrics with smooth surfaces are more suitable for printed circuit fabrication. However, the results of this study demonstrate that even on hydrophilic staple fiber fabric substrates, such as cotton, which have uneven surfaces due to being covered by staple fibers, the electrical performance can be dramatically improved through appropriate mesh density and overprinting, surpassing that of polyester synthetic filament fabrics. This finding contributes to expanding the applicability of printed circuit substrates across a wider range of fabric materials.

## 4. Conclusions

This study systematically analyzed the impact of substrate fabric properties and process conditions on the formation of textile circuits using silver conductive ink screen-printing. While most existing research has focused primarily on fabrics with smooth surfaces and minimal surface roughness, this study specifically evaluated the relationship between mesh density and overprinting conditions on two representative garment materials: hydrophilic cotton staple fiber fabric and hydrophobic polyester filament fabrics.

As the mesh density decreased and the number of print layers increased, the silver conductive layer formed more effectively on the textile substrate, leading to a reduction in the electrical resistance of the printed circuit. In single-layer printing, the porous structure and large surface area of the textile substrate caused high ink absorption, resulting in the silver conductive ink spreading both across the fabric surface and into its interior. This distribution reduced circuit connectivity. However, with repeated printing, enough silver conductive ink was absorbed to build a thicker layer on the surface and improve the connectivity of the conductive material within the fabric. This significantly enhanced the overall circuit connectivity. After three overprinting, the hydrophilic cotton fabric showed lower electrical resistance than the polyester fabric substrate. This is because cotton absorbed the conductive ink more effectively into both its surface and interior. The repeated printing process created a thick silver conductive layer on the surface and established a robust conductive network within the fibers, leading to a substantial improvement in circuit connectivity.

When screen printing on fabric substrates, the bleeding of conductive ink was significantly influenced by mesh density. In the case of hydrophilic cotton fabric substrates, more conductive ink was absorbed internally during the squeegee process compared with the hydrophobic polyester fabric, resulting in less ink bleeding along the length and width of the fabric. To reduce the electrical resistance of the printed circuit, an overprinting method was used, which caused the ink to spread more in both the width and length directions. Therefore, once the substrate fabric to be used and the target electrical resistance of the printed circuit are determined, the optimal screen-printing process conditions should be established based on the findings of this study. Additionally, the dimensional changes due to ink bleeding should be considered when designing circuit patterns.

## Figures and Tables

**Table 1:** Specifications of the silver conductive inks.

Product Name	Appearance	Solids Content (%)	Density (kg/L)	Viscosity mpa.s (cp)	Sheet Resistance (Ω/sq/25 μm)
EDAG 479SS E&C	Silver	75	2.56	12.000	<0.02

**Table 2:** Characteristics of the substrates.

Substrate	Structure	Weight (g/m^2^)	Density		Thickness (mm)
			Warp	Weft	
Cotton fabric	Plain	137.0	136	93	0.21
Polyester fabric	Plain	132.0	199	113	0.20
Polyester film	-	-	-	-	0.10

**Table 3:** Five types of printed circuit design.

Basic	Parallel Straight Lines		Parallel Embattled Lines	
#1	#2	#3	#4	#5
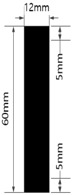	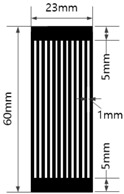	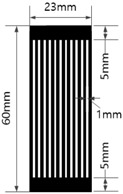	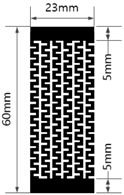	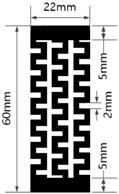

**Table 4:** Microscopic images of the surface of the screen-printed circuits (#1) using silver conductive ink.

Substrate	No. of Print	Mesh Density		
		70G	120G	200G
Polyester film	1	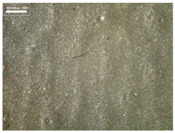	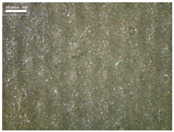	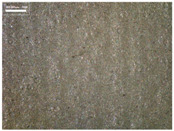
Cotton fabric	1	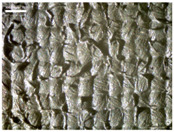	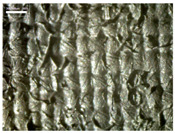	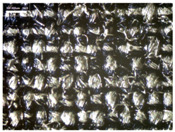
	2	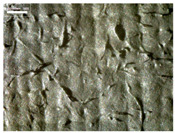	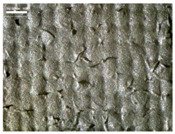	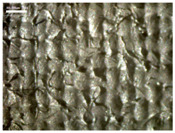
	3	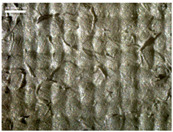	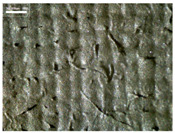	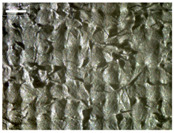
Polyester fabric	1	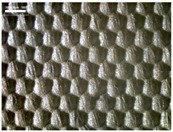	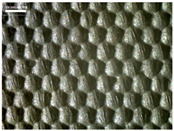	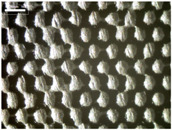
	2	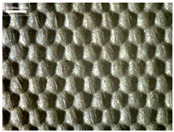	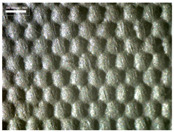	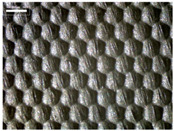
	3	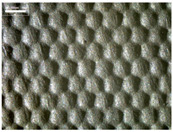	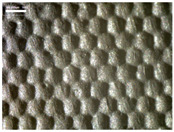	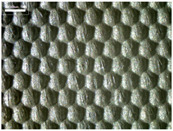

**Table 5:** Scanned images of the surface of the screen-printed circuits (#1) using silver conductive ink.

Substrate	No. of Print	Mesh Density		
		70G	120G	200G
Polyester film	1	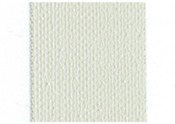	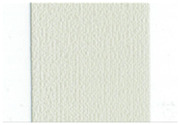	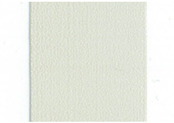
Cotton fabric	1	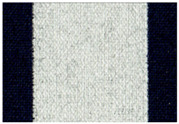	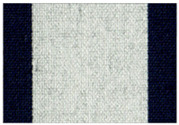	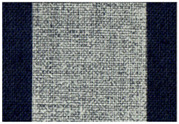
	2	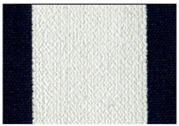	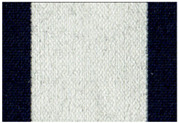	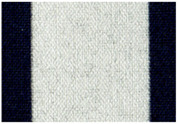
	3	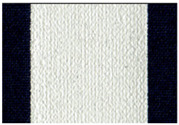	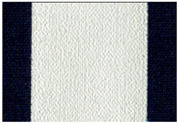	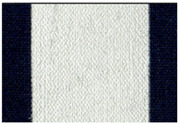
Polyester fabric	1	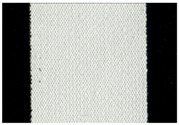	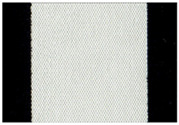	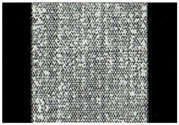
	2	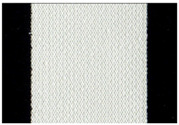	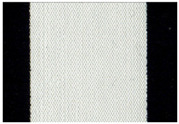	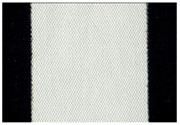
	3	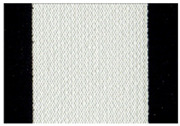	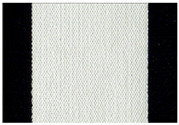	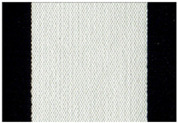

**Table 6:** Cross-sectional SEM images of the two substrates screen-printed with the silver conductive ink.

Substrate	No. of Print	Mesh Density		
		70G	120G	200G
Cotton fabric	1	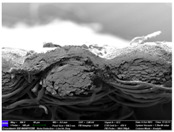	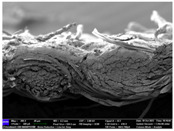	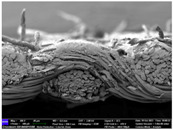
	2	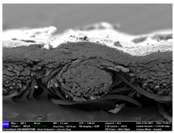	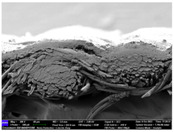	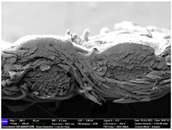
	3	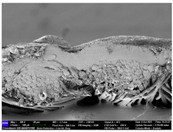	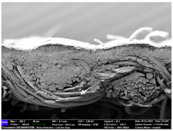	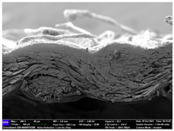
Polyester fabric	1	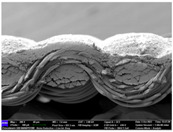	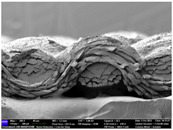	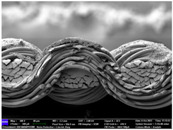
	2	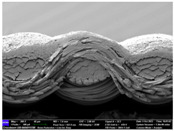	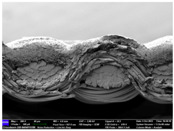	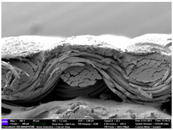
	3	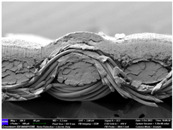	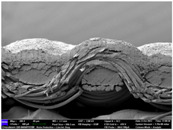	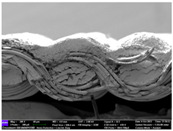

**Table 7:** Scanning images of the textile circuits (#4) screen-printed with the silver conductive ink.

Substrate	No. of Print	Mesh Density		
		70G	120G	200G
Cotton fabric	1	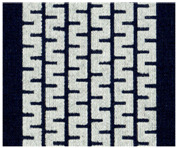	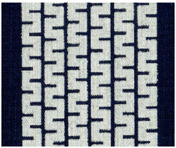	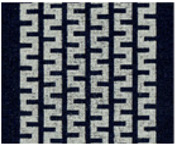
	2	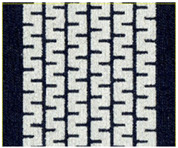	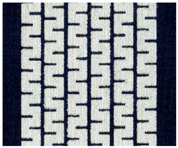	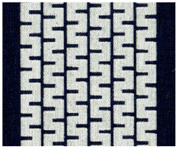
	3	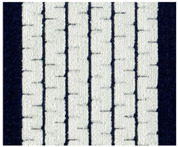	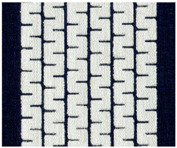	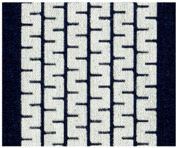
Polyester fabric	1	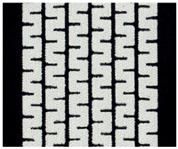	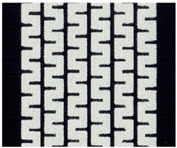	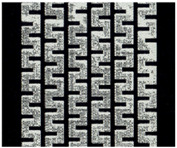
	2	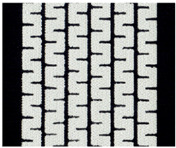	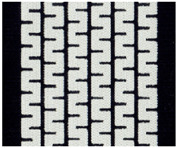	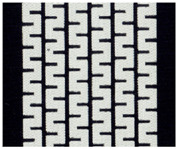
	3	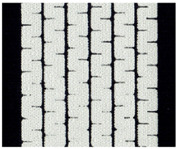	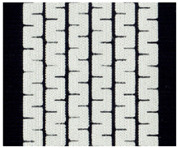	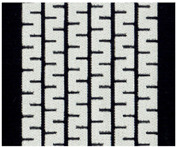

## Data Availability

Data are contained within the article.
